# Pancreas Fat, an Early Marker of Metabolic Risk? A Magnetic Resonance Study of Chinese and Caucasian Women: TOFI_Asia Study

**DOI:** 10.3389/fphys.2022.819606

**Published:** 2022-03-31

**Authors:** Ivana R. Sequeira, Wilson C. Yip, Louise W. W. Lu, Yannan Jiang, Rinki Murphy, Lindsay D. Plank, Garth J. S. Cooper, Carl N. Peters, Jun Lu, Kieren G. Hollingsworth, Sally D. Poppitt

**Affiliations:** ^1^Human Nutrition Unit, Faculty of Science, School of Biological Sciences, University of Auckland, Auckland, New Zealand; ^2^High Value Nutrition National Science Challenge, Auckland, New Zealand; ^3^Department of Statistics, Faculty of Science, University of Auckland, Auckland, New Zealand; ^4^Department of Medicine, Faculty of Medical and Health Sciences, University of Auckland, Auckland, New Zealand; ^5^Auckland District Health Board, Auckland, New Zealand; ^6^Maurice Wilkins Centre for Molecular Biodiscovery, University of Auckland, Auckland, New Zealand; ^7^Department of Surgery, Faculty of Medical and Health Sciences, University of Auckland, Auckland, New Zealand; ^8^Centre for Advanced Discovery and Experimental Therapeutics (CADET), Division of Cardiovascular Sciences, Faculty of Biology, Medicine and Health, University of Manchester, Manchester, United Kingdom; ^9^Faculty of Science, School of Biological Sciences, University of Auckland, Auckland, New Zealand; ^10^Division of Medical Sciences, Department of Pharmacology, University of Oxford, Oxford, United Kingdom; ^11^Waitemata District Health Board, Auckland, New Zealand; ^12^Faculty of Health and Environmental Sciences, Auckland University of Technology, Auckland, New Zealand; ^13^Translational and Clinical Research Institute, Faculty of Medical Sciences, Newcastle University, Newcastle upon Tyne, United Kingdom; ^14^Riddet Centre of Research Excellence for Food and Nutrition, Palmerston North, New Zealand

**Keywords:** liver fat, pancreas fat, visceral adipose tissue, type 2 diabetes, magnetic resonance imaging and spectroscopy

## Abstract

**Objective:**

Prevalence of type 2 diabetes (T2D) is disproportionately higher in younger outwardly lean Asian Chinese compared to matched Caucasians. Susceptibility to T2D is hypothesised due to dysfunctional adipose tissue expansion resulting in adverse abdominal visceral and organ fat accumulation. Impact on early risk, particularly in individuals characterised by the thin-on-the-outside-fat-on-the-inside (TOFI) phenotype, is undetermined.

**Methods:**

Sixty-eight women [34 Chinese, 34 Caucasian; 18–70 years; body mass index (BMI), 20–45 kg/m^2^] from the TOFI_Asia study underwent magnetic resonance imaging and spectroscopy to quantify visceral, pancreas, and liver fat. Total body fat was (TBF) assessed by dual-energy x-ray absorptiometry, and fasting blood biomarkers were measured. Ethnic comparisons, conducted using two-sample tests and multivariate regressions adjusted for age, % TBF and ethnicity, identified relationships between abdominal ectopic fat depots with fasting plasma glucose (FPG), insulin resistance (HOMA2-IR), and related metabolic clinical risk markers in all, and within ethnic groups.

**Results:**

Despite being younger and of lower bodyweight, Chinese women in the cohort had similar BMI and % TBF compared to their Caucasian counterparts. Protective high-density lipoprotein cholesterol, total- and high-molecular weight adiponectin were significantly lower, while glucoregulatory glucagon-like peptide-1 and glucagon significantly higher, in Chinese. There were no ethnic differences between % pancreas fat and % liver fat. However, at low BMI, % pancreas and % liver fat were ∼1 and ∼2% higher in Chinese compared to Caucasian women. In all women, % pancreas and visceral adipose tissue had the strongest correlation with FPG, independent of age and % TBF. Percentage (%) pancreas fat and age positively contributed to variance in FPG, whereas % TBF, amylin and C-peptide contributed to IR which was 0.3 units higher in Chinese.

**Conclusion:**

Pancreas fat accumulation may be an early adverse event, in TOFI individuals, with peptides highlighting pancreatic dysfunction as drivers of T2D susceptibility. Follow-up is warranted to explore causality.

## Introduction

Individuals with obesity have a 50- to 80-fold increased risk of type 2 diabetes (T2D) compared to those with lean bodyweight (Colditz et al., 1995). A decline in lean body mass and consequent increase in body fat often increases with age (Alva et al., 2017), accompanied by altered beta-cell function and insulin resistance (IR). Using body mass index (BMI) as a predictor, however, is shown to potentially misclassify both low BMI (Reilly et al., 2018) and, on occasion, younger (Ramachandran et al., 2012) individuals. Ethnicity is shown to be of importance since, at a fixed BMI, Chinese individuals are shown to have 3–5% higher total body fat (TBF) than Caucasians (Haldar et al., 2015).

Highlighted in large cohort studies (Lear et al., 2007; Nazare et al., 2012; Chen et al., 2018; Jo and Mainous, 2018), including in our recently published TOFI_Asia study (Sequeira et al., 2020), visceral adipose tissue (VAT) is an important factor that differs between these groups. In Chinese, typified by the thin-on-the-outside-fat-on-the-inside (TOFI) phenotype, even modest weight gain may differentially promote lipid deposition into VAT (Hocking et al., 2013) which is associated with increased hepatic IR (Tchernof and Després, 2013), and further promotes ectopic fat infiltration into key metabolic non-adipose tissue organs such as liver and pancreas (Rossi et al., 2011).

The need for phenotypic characterisation based on non-adipose ectopic fat is now recognised to be vital to understanding susceptibility to developing T2D (Neeland et al., 2019), particularly in TOFI individuals (Sequeira et al., 2020). Decreased levels of liver fat are associated with the normalisation of blood glucose (Lim et al., 2011; Nazare et al., 2012; Zhyzhneuskaya et al., 2018) with emerging evidence for the role of pancreas fat (Lim et al., 2011; Taylor et al., 2018). Some studies have reported increased pancreas fat in dysglycaemic individuals (Lingvay et al., 2009; Ou et al., 2013; Gaborit et al., 2014; Kim et al., 2014; Lim et al., 2014; Wang et al., 2014; Macauley et al., 2015; Steven et al., 2016) while others showed a negative correlation with beta-cell function (Tushuizen et al., 2007; Heni et al., 2010; Lim et al., 2011; Szczepaniak et al., 2012; Yokota et al., 2012; Nowotny et al., 2018) albeit as yet inconsistent in cohorts with prediabetes and diabetes (Tushuizen et al., 2007; Lê et al., 2011; van der Zijl et al., 2011). Discrepancies between pancreatic fat and beta-cell dysfunction may be in part due to methodological differences in pancreas fat assessment, in addition to variable glycaemic status in these early studies. A recent meta-analysis attempted to define an upper limit for pancreatic fat percentage, as weighted mean + 2 standard deviations (SDs), determined from pooled data from nine studies in 1,209 healthy individuals who underwent magnetic resonance imaging and spectroscopy (MRI/S) (Singh et al., 2017). A limitation of the recommended normal upper limit of 6.2% pancreas fat, as acknowledged by the authors, are the different methods and quantitative approaches used in the various MR studies which preclude comparisons.

An absence of published data on ectopic fat, particularly pancreatic fat, in different ethnicities, and its contribution to early dysglycaemia led us to undertake the current TOFI_Asia MR study. We hypothesised that fat deposition in non-adipose ectopic sites may be associated with the dysregulation of glucose, and IR, with Chinese participants potentially more susceptible to ectopic lipid infiltration when younger and at lower bodyweight than Caucasians. Furthermore, to identify clinical risk markers, and ectopic fat depots, that are associated with and predictive of fasting plasma glucose (FPG) and IR. Notably, while haemoglobin A1c (HbA1c) is a reliable marker of frank T2D, it has limited sensitivity and specificity for identifying prediabetes (Barry et al., 2017) and hence was not considered an outcome measure.

## Materials and Methods

Ethical approval was received from the New Zealand Health and Disabilities Ethics Committee (16/STH/23) and all research, and study protocols, performed in accordance with Committee guidelines. The study is registered with the Australian New Zealand Clinical Trials Registry ACTRN12616000362493.

### MR Study Population

**FIGURE 1:**
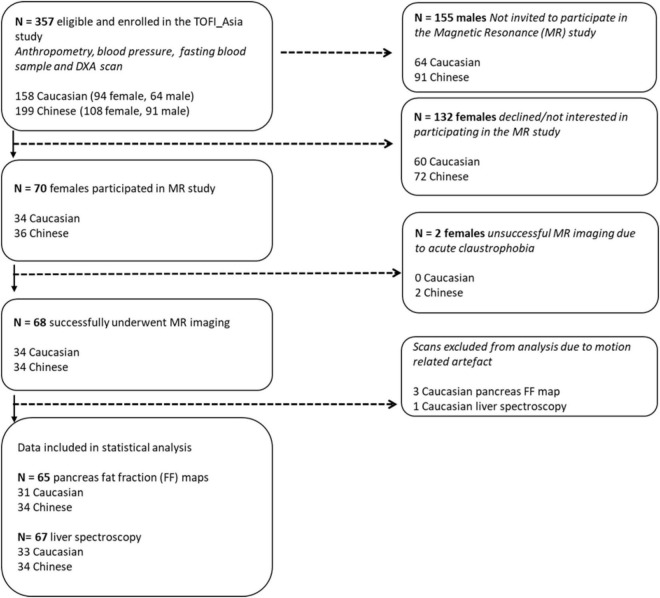
Flowchart illustrating enrolment and eligibility of 202 female participants from the TOFI_Asia study into the MR study. DXA, dual-energy X-ray absorptiometry.

To avoid potential confounding due to gender differences in body composition, women (202; 108 Asian Chinese, 94 European Caucasian) from a larger mixed gender cohort the TOFI_Asia study (Sequeira et al., 2020), recruited in our clinic, were additionally invited for a MRI/S assessment of ectopic visceral, pancreas, and liver fat respectively. The TOFI_Asia cohort consisted of participants over a wide age and BMI range, of which a random cohort of 70 women (20–70 years, BMI 20–45 kg/m^2^) provided informed written consent to participate in the current MR sub-study (Figure 1). Women were normoglycaemic or had impaired fasting glucose (IFG), as defined by the American Diabetes Association (2018), self-reported healthy with no significant disease, no significant weight gain or loss (>10%) in previous 3 months, and no contraindications for MRI/S procedures. Imaging was successfully completed in 68 women (34 Chinese, 34 Caucasian). Two women were excluded due to acute claustrophobic episodes during MRI.

### Experimental Protocol

All women attended the Human Nutrition Unit following an overnight fast. Bodyweight, height, waist/hip circumference, and blood pressure were recorded and fasted blood samples obtained as previously described (Sequeira et al., 2020). TBF was determined by dual-energy X-ray absorptiometry (DXA) (iDXA, GE Healthcare, Madison, WI, United States). MRI/S scans were conducted fasted within 1 week of clinical assessments using a 3T Magnetom Skyra scanner, VE 11A (Siemens, Germany). Instruction for adherence to scan protocol and imaging requirements, e.g., breath hold sequences, were given to minimise motion-related artefact.

#### Analyses of Blood Biochemistry

Venous samples were centrifuged (Eppendorf, HH, Germany) at 1,300 × *g* for 10 min at 4°C with plasma and serum stored at −80°C until batch analyses. HbA1c was determined by capillary electrophoresis (Cap2FP, IDF, France), FPG by the hexokinase method, alanine amino transferase (ALT), aspartate amino transferase (AST), and alkaline phosphatase (ALP) by the International Federation of Clinical Chemistry methods, and gamma glutamyl transferase (GGT) by the Szasz method. Total cholesterol (TC) was analysed by cholesterol esterase/cholesterol oxidase/peroxidase method, triacylglycerol (TAG) by lipase/glycerol kinase method, high-density lipoprotein cholesterol (HDL-C) by detergent/cholesterol esterase/cholesterol oxidase/peroxidase method. Low-density lipoprotein cholesterol (LDL-C) was calculated using the Friedewald formula. Plasma insulin, C-peptide, glucagon, (total) amylin, gastric inhibitory peptide (GIP), and (total) glucagon-like peptide-1 (GLP-1) were analysed using MILLIPLEX MAP Human Metabolic Hormone Magnetic Bead Panel (Merck, HE, Germany). Intra- and inter-assay coefficients of variation (CV) for all analytes were <10 and <15%, respectively. Serum total- and high-molecular weight (HMW) adiponectin was measured by enzyme-linked immunosorbent assay (R&D Systems Quantikine kit, Bio-Techne, Minneapolis, MN, United States). IR, using the updated homeostasis model assessment of insulin resistance (HOMA2-IR), was determined by using the online HOMA Calculator (The University of Oxford 2013, Version 2.2.3).

#### Assessment of Body Composition by Dual-Energy X-Ray Absorptiometry

Total body fat and lean (fat-free soft tissue) masses were obtained from a full body DXA scan with participants measured according to manufacturer’s standard protocols. DXA-percentage (%) TBF was calculated as 100 × TBF mass/(total body lean mass + TBF mass).

#### Assessment of Ectopic Fat by MRI/Magnetic Resonance Spectroscopy

Fast sagittal localising images were acquired from diaphragm to pelvis, using respiratory gating to reduce motion artefacts. A 3D dual gradient-echo sequence (VIBE) was acquired to separate fat and water signals using a 2-point Dixon technique: repetition time/echo times/flip angle = 3.85 ms/1.27, 2.50 ms/9°. Three blocks (11 s breath hold each) of forty 5 mm axial slices using a field of view of 500 mm × 400 mm, matrix 320 × 256, were acquired to cover the abdominal cavity using partial Fourier and parallel imaging with total acceleration factor of 3.1. Following the abdominal scan, the pancreas was located and imaged using a higher resolution sequence, requiring a 11 s breath hold, to acquire fourteen 5 mm axial slices with repetition time/echo times/flip angle/signal averages = 5.82 ms/2.46, 3.69 ms/9°/1 with a field of view of 500 mm × 400 mm, matrix 512 × 410, using partial Fourier and parallel imaging with total acceleration factor of 2.8. MRS with respiratory-gated sequence determined liver fat content. Localiser images were obtained in the transverse, coronal, and sagittal planes and a voxel (2 × 2 × 2 cm^3^) placed in the right lobe of the liver, avoiding blood vessels and the biliary tree. MRS of the selected voxel was acquired using the stimulated-echo acquisition mode sequence with respiratory triggering; echo time: 20 ms, repetition time: 3,000 ms and mixing time: 33 ms, 1,024 data points collected with 50 averages. A water-suppressed spectrum with 50 averages was also recorded to detect weak lipid signals.

#### Magnetic Resonance Imaging and Spectroscopy Image Analysis

Custom Matlab R2017a software (The MathWorks, Inc., Natick, MA, United States) was used to separate the fat and water contributions of the abdominal MRI and construct a fat fraction (FF) map with noise bias correction at the L4–L5 intervertebral disc space. The single FF map was separated into MR-VAT and MR-subcutaneous adipose tissue (SAT) with Image J (Schneider et al., 2012), using the polygon tool to manually circumscribe contours around each region. The single slice MR abdominal adipose tissue (MR-AAT) measurement at the L4–L5 anatomical location is shown to be concordant with fat mass measured at all intervertebral spaces (Schweitzer et al., 2015; Linder et al., 2016). MRI analysis was conducted independently by two trained investigators, IS and WY. The intra-observer repeatability CV for MR-VAT and MR-SAT, conducted by IS calculated from 10% of total scans was 0.2 and 0.1%, respectively, while the inter-observer CV of all scans, between IS and WY, was 0.02 and 0.1%. Percentage (%) pancreas fat was determined by MR-opsy (Al-Mrabeh et al., 2017) with thresholding applied to eliminate potential inclusion of non-parenchymal tissue. Briefly, two candidate pancreas (5 mm each) FF maps were created from images in which the head, body, and tail of the pancreas were clearly visualised. Three regions of interest (ROIs) were placed in the head, body, and tail of each image, respectively, to estimate pancreas fat; thresholding (1–20%) was also applied to eliminate potential inclusion of non-parenchymal tissue within the selected ROI. Percentage pancreas fat was calculated as the average fat of both candidate pancreas FF images [39]. FF maps obtained from three Caucasian women contained artefact and were unable to be analysed, hence % pancreas fat was measured in 65 women. As conducted for MR-AAT compartments, the intra-observer repeatability % pancreas fat CV, from 10% of total scans, was 3.4% and the inter-observer CV was 2.4%. Pancreas volume (cm^3^) was determined as previously described (Macauley et al., 2015), and volume divided by body surface area, using the Dubois and Dubois method, to obtain pancreas volume index (PVI) which accounts for potential effect of anthropometry on measurements. Liver fat was calculated, using the SIVIC software (Crane et al., 2013), from area under the curve of water and fat peaks from non-water-suppressed spectra, corrected for T2-weighting according to previous literature values (Hamilton et al., 2011) and presented as percentage volume/volume from 67 women; the spectroscopy signal obtained from one Caucasian woman could not be analysed. Liver fat ≥5.6% was considered elevated, this cut off reported as the upper 95 percentile in healthy subjects that corresponds to ∼15% histological liver fat (Petäjä and Yki-Järvinen, 2016). There is no global comparable cut off for % pancreas fat, particularly since our pancreas imaging in this study has T1 weighting and will overestimate FF by a factor of approximately 1.4 (de Bazelaire et al., 2004). To internally compare % pancreas fat in our cohort of women we used an arbitrary 4.5% cut off.

### Statistical Analyses

Continuous variables are expressed as mean ± SD in the descriptive summary. Group comparisons were performed using two-sample tests with level of significance set at *P* < 0.05. The distribution of outcome measures (FPG and HOMA2-IR) was assessed and log-transformation was applied to non-normally distributed data if applicable. Relationships between the two outcome measures with MR-VAT (cm^2^), % pancreas and % liver fat were assessed using multiple linear regression models in all women and for each ethnic group separately. The models were adjusted for age and % TBF, as well as ethnicity in the total cohort. The interaction effect between ethnicity and the predictor of interest was tested in the models to assess ethnicity as a potential effect modifier. All MR quantified non-adipose tissue organ fat measurements were included in statistical analyses, as no outliers were detected. Of the two outcome variables, analyses were conducted on raw FPG data while HOMA2-IR was normalised using log-transformation due to skewed distribution. Univariate and multivariate regression analyses were conducted to identify clinical risk markers that predicted the difference in FPG and log HOMA2-IR. Associations between the clinical risk markers and FPG, and log HOMA2-IR, were each assessed using single predictor linear regression models, adjusted for ethnicity. Those clinical risk markers that showed statistical significance at *P* < 0.1 were included in stepwise multiple linear regression analyses using SAS version 9.4 (SAS Institute Inc., Cary, NC, United States). The software utilises a combination of backward and forward selection techniques to retain independent predictors that showed a significant effect (*P* < 0.05) to give a final prediction model with risk markers that predicted FPG, and log HOMA2-IR. Additionally, we also performed stepwise multiple linear regression and the least angle regression (LAR) models treating ethnicity as a potential predictor same as the clinical markers that showed statistical significance at *P* < 0.1 in the models. The results were cross-validated with the main analysis using ethnicity-adjusted stepwise regression models.

## Results

### Participant Characteristics

Mean age and BMI of the full cohort was 44.4 ± 14.5 years and 27.3 ± 4.4 kg/m^2^. The women were predominantly healthy and normoglycaemic, with 19% (*n* = 13) with IFG with an equal division of prediabetes between ethnicities (*n* = 7 Caucasian, *n* = 6 Chinese). Although significantly younger (*P* = 0.05), the Chinese women were of similar mean BMI to Caucasian (*P* > 0.05, Table 1) with lower mean bodyweight, height (both *P* < 0.001), waist (*P* = 0.05), and hip circumference (*P* = 0.002).

**TABLE 1:** Summary of metabolic risk factors of women enrolled in the TOFI_Asia MR study.

	All (*n* = 68)	Chinese (*n* = 34)	Caucasian (*n* = 34)	*P* value
Age (year)	44.4 ± 14.5	41.0 ± 13.0	47.8 ± 15.4	0.05
**Anthropometry**				
Bodyweight (kg)	73.7 ± 13.9	68.5 ± 11.5	79.0 ± 14.2	0.001
Height (m)	1.64 ± 0.08	1.60 ± 0.05	1.68 ± 0.08	<0.0001
BMI (kg/m^2^)	27.3 ± 4.4	26.7 ± 4.2	28.0 ± 4.5	0.24
Waist circumference (cm)	88.6 ± 12.9	85.6 ± 11.1	91.7 ± 13.9	0.05
Hip circumference (cm)	101.0 ± 11.8	96.8 ± 9.0	105.3 ± 12.8	0.002
Systolic blood pressure, SBP (mmHg)	120 ± 21	120 ± 22	120 ± 19	0.93
Diastolic blood pressure, DBP (mmHg)	64 ± 10	65 ± 12	64 ± 8	0.61
**Body composition – DXA**				
Total body fat, DXA-TBF (kg)	29.0 ± 10.0	26.2 ± 7.2	31.8 ± 11.7	0.02
Total body fat, DXA-TBF (%)	39.8 ± 7.6	39.2 ± 4.9	40.4 ± 9.6	0.51
**Body composition – MRI/S**				
Visceral adipose tissue, MR-VAT (cm^2^)	73.2 ± 38.8	70.2 ± 31.2	76.3 ± 45.4	0.52
Subcutaneous adipose tissue, MR-SAT (cm^2^)	165.3 ± 65.1	138.7 ± 30.6	191.9 ± 78.8	0.001
Abdominal adipose tissue, MR-AAT (cm^2^)	238.5 ± 87.4	208.9 ± 48.7	268.2 ± 106.5	0.004
VAT:SAT ratio	0.445 ± 0.3	0.51 ± 0.2	0.398 ± 0.3	0.46
Pancreas fat (%)^a^	4.2 ± 1.9	4.3 ± 2.0	4.1 ± 1.9	0.69
Liver fat (%)^b^	4.2 ± 4.8	4.6 ± 4.7	3.7 ± 4.8	0.47
Pancreas volume (cm^3^)^a^	74.0 ± 19.6	72.9 ± 22.3	75.2 ± 16.4	0.63
Pancreas volume index, PVI^a^	41.5 ± 10.7	42.4 ± 12.0	40.6 ± 9.1	0.49
**Blood biochemistry**				
HbA1c (mmol/mol)	34.7 ± 3.9	35.5 ± 3.8	33.9 ± 3.9	0.09
Fasting plasma glucose, FPG (mmol/L)	5.1 ± 0.6	5.2 ± 0.5	5.1 ± 0.7	0.52
Fasting plasma insulin (pg/ml)	537.7 ± 363.1	578.1 ± 350.3	497.4 ± 376.4	0.36
HOMA2-IR	1.7 ± 1.1	1.8 ± 1.0	1.6 ± 1.2	0.37
HOMA2-β	129.9 ± 68.3	138.4 ± 78.3	121.4 ± 56.3	0.31
Total cholesterol, TC (mmol/L)	4.8 ± 0.9	4.5 ± 0.9	5.2 ± 0.9	0.004
Triglycerides, TAG (mmol/L)	1.1 ± 0.6	1.3 ± 0.7	1.0 ± 0.5	0.09
HDL-cholesterol (mmol/L)	1.6 ± 0.4	1.4 ± 0.4	1.8 ± 0.4	0.001
LDL-cholesterol (mmol/L)	2.7 ± 0.8	2.5 ± 0.7	2.9 ± 0.9	0.02
TC:HDL-C ratio	3.2 ± 0.8	3.3 ± 0.8	3.0 ± 0.8	0.38
Alanine amino transferase, ALT (U/L)	14.9 ± 10.1	14.7 ± 10.7	15.1 ± 9.6	0.88
Aspartate amino transferase, AST (U/L)	18.8 ± 7.4	17.8 ± 5.4	19.9 ± 90	0.24
Alkaline phosphatase, ALP (U/L)	96.6 ± 27.4	92.9 ± 26.0	100.4 ± 28.6	0.27
Gamma glutamyl transferase, GGT (U/L)	23.1 ± 20.7	26.3 ± 27.1	20.0 ± 10.5	0.21
Amylin (pg/mL)	29.1 ± 13.7	29.9 ± 13.8	28.3 ± 13.8	0.63
C-peptide (pg/mL)	950.9 ± 502.6	918.5 ± 400.0	983.4 ± 592.2	0.60
Gastric inhibitory peptide, GIP (pg/mL)	68.7 ± 37.9	71.5 ± 40.8	66.0 ± 35.2	0.55
Glucagon like peptide-1, GLP-1 (pg/mL)	136.2 ± 45.5	147.6 ± 46.2	124.9 ± 42.4	0.04
Glucagon (pg/mL)	57.9 ± 32.0	65.6 ± 33.1	50.1 ± 29.5	0.05
Total adiponectin (mg/L)	8.5 ± 5.2	6.8 ± 4.9	10.2 ± 5.0	0.006
HMW adiponectin (mg/L)	6.1 ± 3.9	4.8 ± 3.6	7.5 ± 3.9	0.005

*Results are mean ± SD. Statistical significance P < 0.05. Numbers as stated above each column except for superscripted values.*

*^a^Pancreas fat assessed in 31 Caucasian women.*

*^b^Liver fat assessed in 33 Caucasian women.*

*DXA, dual-energy x-ray absorptiometry; MRI/S, magnetic resonance imaging and spectroscopy; HbA1c, haemoglobin A1c; HOMA2-IR, homeostasis model assessment of insulin resistance; HOMA2-β, homeostasis model assessment of β-cell function; HMW, high-molecular weight.*

### Body Composition—Adipose Tissue Compartments

**FIGURE 2:**
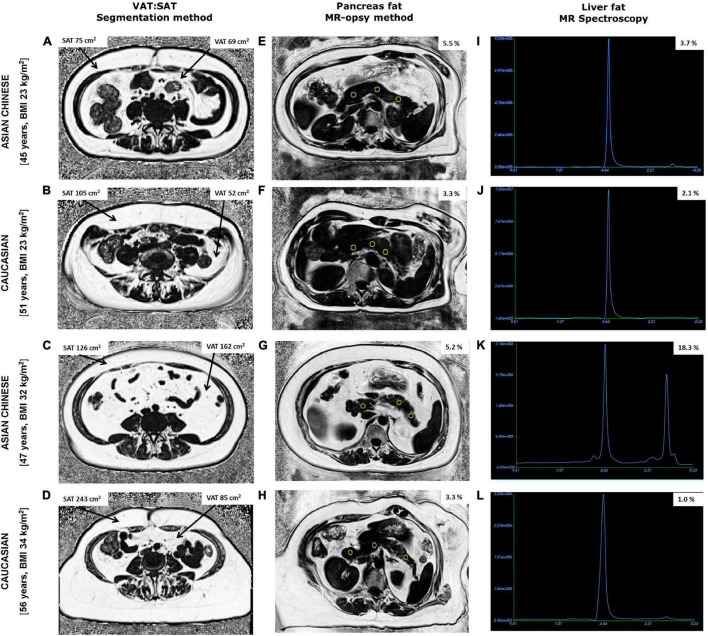
Fat fraction maps obtained from abdominal MRI scans showing subcutaneous (SAT), visceral adipose tissue (VAT), and pancreas fat along with MR spectroscopy of liver fat from two Chinese and two Caucasian women. Chinese women aged 45 years, BMI 23 kg/m^2^ **(A,E,I)** and 47 years, BMI 32 kg/m^2^ **(C,G,K)** had lower SAT and higher pancreas fat compared to older BMI matched Caucasian women aged 51 years, BMI 23 kg/m^2^ **(B,F,J)** and 56 years, BMI 34 kg/m^2^ **(D,H,L)**, respectively. Notably, VAT, % pancreas and % liver fat were higher in both Chinese women compared with BMI matched Caucasian counterparts exhibiting the TOFI profile.

Mean DXA-% TBF was 39.8 ± 7.6% in the full cohort of women. In accordance with lower bodyweight and stature, DXA-assessed TBF mass (kg) was also significantly lower in Chinese (*P* = 0.02) but when normalised as % of total soft tissue mass there were no ethnic-specific differences in DXA-% TBF between the Chinese and Caucasian women (Table 1). While MR-assessed SAT (cm^2^) and MR-VAT (cm^2^) were again both numerically lower in these smaller stature Chinese women, notably this difference was highly significant for MR-SAT (*P* = 0.001, Figure 1) but not for MR-VAT (*P* > 0.05) which was not significantly different from the Caucasian subcohort. In turn, while mean MR-AAT (cm^2^) was again significantly lower (*P* = 0.004) in smaller stature Chinese than Caucasian women, there was greater contribution of VAT (34 vs. 28%) than SAT (66 vs. 72%) to the abdominal fat compartment in the Chinese subcohort (Figure 2).

### Non-adipose Tissue Organ Fat—Pancreas and Liver

Mean MRI-assessed % pancreas fat (determined in 65 of 68 women) was 4.2 ± 1.9% (Table 1). Mean pancreas volume was 74.0 ± 19.6 cm^3^, and PVI was 41.5 ± 10.7. Notably, 39% (*n* = 25; mean age: 49 ± 12 years; BMI: 28.7 ± 4.4 kg/m^2^) of women were identified with pancreas fat above our chosen threshold of 4.5% with mean pancreas volume and PVI of 77.3 ± 18.6 cm^3^ and 41.5 ± 9.6, respectively. Similarly, mean MRS-assessed % liver fat (measured in 67 of 68 women) was lower than the 5.6% threshold (mean: 4.2 ± 4.8%, Table 1) with 22% (*n* = 15; mean age: 50.1 ± 13.0 years; BMI: 28.9 ± 3.6 kg/m^2^) of women identified with elevated liver fat.13% of women (*n* = 9, mean age: 52.8 ± 12.2 years; BMI: 29.4 ± 3.9 kg/m^2^) had both fatty liver and pancreatic fat above the chosen cut offs.

Comparisons of organ fat between the two ethnic groups showed no significant difference in mean % pancreas fat (*P* > 0.05, Table 1). However, linear regression (Supplementary Figure 1) revealed that at a fixed BMI in the lean range between 20 and 25 kg/m^2^ Chinese women had ∼1% higher predicted pancreas fat than their Caucasian counterparts (range: 3–3.5 vs. 1.8–2.7%). Notably, there were similar numbers of Chinese (*n* = 13) and Caucasian (*n* = 12) women with % pancreas fat above the chosen threshold, despite the Chinese subcohort being 2 BMI units lower (mean BMI: 27.9 ± 5.2 vs. 30.0 ± 3.3 kg/m^2^; *P* = 0.35) and 10 years younger (mean age: 44 ± 11 vs. 54 ± 12 years; *P* = 0.06) than Caucasian. Mean pancreas volume and mean PVI were not significantly different between ethnicities in the full cohort (Table 1). Also similarly, no difference in either pancreas volume (Chinese: 68.1 ± 20.5 cm^3^; Caucasian: 77.3 ± 18.6 cm^3^) or PVI (Chinese: 38.4 ± 9.6; Caucasian: 41.5 ± 9.6) was observed in Chinese and Caucasian women with pancreas fat above the chosen 4.5% threshold.

Again, while there was no significant difference in mean % liver fat between the two ethnicities, linear regression analysis showed, as for % pancreas fat, that at a fixed BMI in the lean range of between 20 and 25 kg/m^2^ (Supplementary Figure 1) Chinese women had ∼2% higher predicted liver fat than their Caucasian counterparts (range: 3–3.5 vs. 1.3–1.5%). Twice as many Chinese (*n* = 10) had elevated liver fat (Caucasian, *n* = 5), despite the Chinese subcohort being 3 BMI units lower (mean BMI: 27.9 ± 4.9 vs. 30.8 ± 3.5 kg/m^2^; *P* = 0.10) and 10 years younger (mean age: 44 ± 12 vs. 55 ± 12 years; *P* = 0.03) than their Caucasian counterparts.

### Blood Biochemistry

Mean concentrations of all blood biochemical markers were within the normal range in the full cohort. However, TC (*P* = 0.004), LDL-C (*P* = 0.02), HDL-C (*P* = 0.001), total- (*P* = 0.006), and HMW adiponectin (*P* = 0.005) were significantly lower, while GLP-1 (*P* = 0.04) and glucagon (*P* = 0.05) were significantly higher, in Chinese compared to Caucasian women.

### Relationship Between Metabolic Risk Factors

Associations between MR-VAT, % pancreas and % liver fat depots with FPG and log HOMA2-IR were strengthened by age and % TBF, and independent of ethnicity. The adjusted models showed that despite significant improvement in the relationships with addition of these covariates, the variance, i.e., beta coefficient, attributed to the depots themselves weakened to non-significance in the overall relationships (Table 2 and Supplementary Table 1). FPG was significantly and positively associated with MR-VAT (*r* = 0.47, *P* < 0.0001) and notably % pancreas fat (*r* = 0.46, *P* < 0.0001) in the full cohort, unlike % liver fat (*r* = 0.21, *P* = 0.09) (Supplementary Figure 2). While both MRI measurements had the strongest correlation with FPG, these relationships were strengthened when linear models were adjusted for age, % TBF and ethnicity (both, *r* = 0.54, *P* < 0.0001). Interestingly, it was age that explained the most significant variance in FPG in both the adjusted models. Although non-significant for MR-VAT (Table 2) the beta coefficient between % pancreas fat and FPG (Table 2) remained statistically significant in all women. The adjusted model improved the relationship between % liver fat with FPG (*r* = 0.54, *P* = 0.009) with the most significant independent predictors being age and % TBF in the full cohort (Supplementary Table 1). Again, the only significant positive relationship with log HOMA2-IR was observed in the full cohort with MR-VAT (*r* = 0.24, *P* = 0.05). Adjusted models, however, significantly improved relationships with IR and expectedly were mainly driven by % TBF (Supplementary Table 1).

**TABLE 2:** Relationships between fasting plasma glucose, and insulin resistance (log HOMA2-IR) with visceral adipose tissue, pancreas, and liver fat in the MR cohort.

	FPG (mmol/L)					log HOMA2-IR				
	β	SE	*t*	*P* value	Interaction with ethnicity, *P* value	β	SE	*t*	*P* value	Interaction with ethnicity, *P* value
Visceral adipose tissue, MR-VAT (cm^2^)					0.30					0.28
All	0.003	0.003	1.15	0.25		0.002	0.003	0.73	0.47	
Chinese	−0.001	0.004	−0.14	0.89		0.002	0.004	0.49	0.63	
Caucasian	0.006	0.004	1.40	0.17		0.001	0.005	0.15	0.88	
Pancreas fat (%)					0.76					0.19
All	0.08	0.04	1.99	0.05		0.008	0.05	0.16	0.87	
Chinese	0.07	0.04	1.64	0.11		−0.01	0.05	−0.30	0.77	
Caucasian	0.10	0.09	1.15	0.26		0.03	0.11	0.30	0.77	
Liver fat (%)					0.25					0.09
All	−0.005	0.015	−0.295	0.77		−0.006	0.02	−0.35	0.72	
Chinese	−0.02	0.02	−1.05	0.30		−0.03	0.02	−1.14	0.26	
Caucasian	0.01	0.02	0.42	0.68		0.01	0.03	0.40	0.69	

*Data are presented as beta coefficients for each metabolic risk factor with FPG and log HOMA2-IR. All models are adjusted for age and % total body fat. The models for all women also additionally adjusted for ethnicity. The interaction effect between ethnicity and the predictor of interest was tested. Statistical significance at P < 0.05. Pancreas fat determined from MRI scans in 65 women (31 Caucasian and 34 Chinese); liver fat determined from MRS scans in 67 women (33 Caucasian and 34 Chinese).*

### Independent Predictors of Fasting Plasma Glucose and Insulin Resistance

Of the clinical risk markers, age and % pancreas fat positively, while liver enzyme AST negatively, contributed to the variance in the model for predicting FPG (*P* < 0.0001) (Table 3, with all significant independent predictors Table 4). Notably, ethnicity did not contribute to the model (*P* = 0.20). On the contrary, ethnicity explained the greatest variance in the model for IR (log HOMA2-IR), which was higher in Chinese, followed by % TBF, amylin and C-peptide concentrations (*P* < 0.0001) (Table 3).

**TABLE 3:** Stepwise linear regression models, adjusted for ethnicity, with significant independent metabolic risk factors that predict (i) fasting plasma glucose and (ii) insulin resistance (log HOMA2-IR) in women (*n* = 68) from the MR study.

	β coefficient	SE	*t*	*P* value
**FPG (mmol/L)**				
Intercept	4.42	0.27	16.44	<0.001
Chinese	0.16	0.13	1.28	0.20
Age	0.01	0.005	2.58	0.01
Pancreas fat (%)	0.09	0.04	2.60	0.01
AST (U/L)	−0.02	0.008	−2.35	0.02
**log HOMA2-IR**				
Intercept	−1.45	0.31	−4.75	<0.001
Chinese	0.30	0.11	2.71	0.009
DXA-%TBF	0.02	0.008	2.49	0.02
Amylin (pg/ml)	0.01	0.006	2.14	0.04
C-peptide (pg/ml)	0.001	0.0001	3.57	0.001

*Summary of the Stepwise models are as follows (i) FPG: R^2^ = 0.33, P < 0.0001 and (ii) log HOMA2-IR: R^2^ = 0.56, P < 0.0001. Models for each outcome include all independent significant predictors (P < 0.10); details available in Table 4. Statistical significance was set at P < 0.05.*

**TABLE 4:** Association between (i) fasting plasma glucose (FPG) and (ii) insulin resistance (calculated using HOMA2-IR) with individual metabolic risk factors in *n* = 68 women that underwent MR imaging using single predictor regression models.

	FPG (mmol/L)			log HOMA2-IR		
	Beta coefficients (±95% CI)	*R* ^2^	*P* value	Beta coefficients (±95% CI)	*R* ^2^	*P* value
Age (year)	0.02 (0.01, 0.03)	0.20	0.0002	−0.003 (−0.01, 0.01)	0.04	0.66
BMI (kg/m^2^)	0.04 (0.003, 0.07)	0.07	0.03	0.06 (0.03, 010)	0.21	0.001
Waist circumference (cm)	0.01 (0.003, 0.03)	0.09	0.02	0.02 (0.001, 0.03)	0.15	0.006
Systolic blood pressure, SBP (mmHg)	0.01 (0.004, 0.02)	0.14	0.002	0.01 (−0.003, 0.01)	0.06	0.20
Diastolic blood pressure, DBP (mmHg)	0.01 (−0.002, 0.03)	0.05	0.09	0.01 (−0.01, 0.02)	0.06	0.28
Total body fat, DXA-TBF (kg)	0.01 (−0.002, 0.03)	0.05	0.09	0.03 (0.01, 0.04)	0.21	0.0003
Total body fat, DXA-TBF (%)	0.03 (0.008, 0.05)	0.12	0.006	0.04 (0.02, 0.06)	0.23	0.0002
Visceral adipose tissue, MR-VAT (cm^2^)	0.007 (0.004, 0.01)	0.23	0.001	0.004 (0.0003, 0.008)	0.10	0.03
Pancreas fat (%)	0.14 (0.07, 0.20)	0.22	0.0002	0.05 (−0.03, 0.13)	0.07	0.27
Liver fat (%)	0.01 (−0.002, 0.03)	0.05	0.09	0.01 (−0.01, 0.02)	0.07	0.41
Fasting plasma insulin (pg/mL)	0.0003 (−0.0001, 0.001)	0.03	0.18	0.002 (0.001, 0.002)	0.82	<0.0001
Total cholesterol (mmol/L)	0.02 (−0.15, 0.19)	0.01	0.83	−0.05 (−0.23, 0.13)	0.04	0.56
Triglycerides, TAG (mmol/L)	0.25 (0.01, 0.50)	0.07	0.04	0.24 (−0.02, 0.50)	0.09	0.07
HDL-cholesterol (mmol/L)	−0.27 (−0.64, 0.10)	0.04	0.14	−0.52 (−0.90, −0.14)	0.14	0.001
LDL-cholesterol (mmol/L)	0.03 (−0.17, 0.23)	0.01	0.76	0.01 (−0.20, 0.22)	0.04	0.94
Alanine amino transferase, ALT (U/L)	0.001 (−0.01, 0.02)	0.01	0.91	0.001 (−0.02, 0.02)	0.04	0.94
Aspartate amino transferase, AST (U/L)	−0.02 (−0.04, 0.001)	0.06	0.06	−0.02 (−0.04, 0.002)	0.09	0.07
Alkaline phosphatase, ALP (U/L)	0.002 (−0.003, 0.008)	0.02	0.40	0.001 (−0.005, 0.007)	0.04	0.65
Gamma glutamyl transferase, GGT (U/L)	0.000 (−0.007, 0.007)	0.01	0.99	0.0002 (−0.008, 0.008)	0.04	0.95
Amylin (pg/mL)	0.01 (−0.001, 0.02)	0.05	0.07	0.03 (0.02, 0.04)	0.40	<0.0001
C-peptide (pg/mL)	0.001 (0.0002, 0.001)	0.15	0.001	0.001 (0.001, 0.001)	0.47	<0.0001
Gastric inhibitory peptide, GIP (pg/mL)	−0.001 (−0.005, 0.003)	0.01	0.71	0.004 (0.0003, 0.01)	0.10	0.03
Glucagon like peptide-1, GLP-1 (pg/mL)	−0.001 (−0.005, 0.002)	0.02	0.43	0.01 (0.003, 0.01)	0.23	0.0001
Glucagon (pg/mL)	−0.002 (−0.01, 0.003)	0.01	0.50	0.007 (0.002, 0.01)	0.14	0.007
Total adiponectin (mg/L)	0.0001 (−0.03, 0.03)	0.006	0.99	−0.02 (−0.05, 0.02)	0.06	0.30
High-molecular weight adiponectin (mg/L)	0.005 (−0.04, 0.05)	0.007	0.81	−0.03 (−0.07, 0.02)	0.06	0.21

*Data are presented as beta coefficients (±95% confidence intervals) for each metabolic risk factor with (i) FPG and (ii) log HOMA2-IR. Statistical significance at P < 0.10. Each model is adjusted for Ethnicity*

## Discussion

In the TOFI_Asia MR cohort, comprising healthy and dysglycaemic Chinese and Caucasian women with similar BMI and % TBF, we show the importance of phenotypic characterisation of MRI/S quantified ectopic fat and highlight the need for early markers of T2D risk, aligning with and in accordance to the recent joint position statement from the International Atherosclerosis Society and the International Chair on Cardiometabolic Risk Working Group on Visceral Adiposity (Neeland et al., 2019). Multivariate analysis revealed MRI-assessed % pancreas fat as a positive predictor of increased FPG, and pancreatic glucoregulatory peptides amylin, that is involved in muscle glycogenolysis (Young et al., 1993), and C-peptide, a component of proinsulin, as predictors of IR. Additional linear relationships with non-adipose % pancreas fat suggest that it may be an early T2D risk marker, particularly in Chinese characterised by TOFI, which contributes to increased IR and/or declining beta-cell function to alter glucose metabolism and requires further investigation.

Recent studies have demonstrated ethnic variability in abdominal and visceral adiposity (Lear et al., 2007; Nazare et al., 2012; Chen et al., 2018), including the wider TOFI_Asia study (Sequeira et al., 2020), and shown increased risk of dysglycaemia associated with these compartments. Although accumulation of pancreas fat is postulated to result in damage to the beta-cell in preclinical studies (Lee et al., 2009), causal associations with beta-cell dysfunction, and glucose intolerance in humans remains to be established. The pancreas plays a key role in T2D, characterised by chronic hyperglycaemia in the context of IR and/or beta-cell dysfunction. Insulin secretion (IS) in response to a glucose challenge is complex and dependent not only on co-existing plasma glucose concentration but also beta-cell responsiveness to changing glucose levels and the rate of insulin clearance, both modulated by the prevailing IR (Del Prato et al., 2002). In IR, increased beta-cell response to glucose and reduced insulin clearance are adaptive mechanisms to maintenance normal glucose tolerance.

Previous work in dysglycaemic individuals, including Chinese and Caucasians, has reported increased pancreas fat (Lingvay et al., 2009; Ou et al., 2013; Gaborit et al., 2014; Kim et al., 2014; Lim et al., 2014; Wang et al., 2014; Macauley et al., 2015; Steven et al., 2016) and decreased beta-cell function (Tushuizen et al., 2007; Heni et al., 2010; Lim et al., 2011; Szczepaniak et al., 2012; Yokota et al., 2012). There are, however, inconsistent findings regarding the association between pancreatic fat and glucose metabolism (Tushuizen et al., 2007; Lê et al., 2011; van der Zijl et al., 2011), possibly due to varied methodologies in determination of pancreas fat and/or the heterogeneity in distribution of fat within the pancreas itself (Schwenzer et al., 2008; Lee et al., 2009; Smits and van Geenen, 2011) which often precludes comparisons between studies. In our current study we adopted the MR-opsy method (Al-Mrabeh et al., 2017), and though mean % pancreas fat was below the threshold adopted for internal comparisons between ethnicities in our cohort, a wide range was observed with pancreas fat an important predictor of FPG compared to the other adipose tissue compartments and liver. There were no ethnic differences observed in mean pancreas volume or index; however, the mean levels observed were concordant to those previously reported (Macauley et al., 2015) in healthy normoglycaemic individuals.

Furthermore, a significant association between FPG with pancreas and liver fat in the cohort was observed, notably with some participants with raised FPG (≥5.6 mmol/L) even when fat infiltration in these organs was low to possibly demonstrate that even small amounts of ectopic fat, likely representative of early stage deposition, may increase T2D susceptibility. Intrahepatic fat inhibits insulin suppression of hepatic glucose output to consequently result in increased basal IS, which exacerbates liver fat content and increases circulating plasma TAG concentration. Exposure of pancreatic beta-cells to excess fatty acids, derived from circulating and locally deposited TAG, inhibits glucose-mediated IS with consequent increase in plasma glucose. The degree of beta-cell decompensation is thought to occur based on a personal fat threshold which when exceeded exacerbates T2D risk (Taylor and Holman Rury, 2015).

Alongside % pancreas fat and age, lower concentration of AST predicted higher FPG in this MR study. Elevated liver function enzymes ALT and GGT, but not AST, have previously been associated with increased risk of T2D in meta-analyses (Fraser et al., 2009; Kunutsor et al., 2013). However, AST, like ALT, catalyses the transfer of amino groups to generate by-products during gluconeogenesis and amino acid metabolism, so it is not unexpected that lower concentrations may be associated with variable FPG. The concordance of findings reiterated in LAR and Stepwise regression analyses without ethnicity included in the model (Supplementary Tables 2A,B) additionally emphasises that % pancreas fat is indeed a strong predictor of FPG. Furthermore, significant variability in IR in the multiple regression models (also concordant with results from Supplementary Tables 2A,B) was explained by % TBF, pancreatic glucose regulating peptides amylin and C-peptide. While amylin decreases glucagon-stimulated hepatic glucose output, and shown to regulate muscle glycogenolysis, this does not occur during insulin-induced hypoglycaemic states (Aronoff et al., 2004). It is notable that the most significant contribution to variation in IR was ethnicity, with HOMA2 assessed IR greater by 0.3 units in the Chinese.

Phenotypic characterisation also revealed that Chinese women in the cohort although younger, and of lower bodyweight and stature, had similar BMI, % TBF, MR-VAT (cm^2^) and non-adipose tissue % pancreas and % liver fat than the Caucasian women. Conversely, abdominal SAT (cm^2^) was lower. Outwardly lean, low BMI Chinese women were also observed with greater propensity to TOFI than their lean Caucasian counterparts, with fixed BMI cut-points of 20 and 25 kg/m^2^ associated with greater pancreas fat and greater liver fat content in the Chinese subcohort. Also, lower protective circulating HDL-C and glucose regulating total- and HMW adiponectin were observed in Chinese than Caucasian women. Circulating peptides GLP-1 and glucagon, also involved in glucose regulation, were also higher. Although not possible to draw conclusions from this smaller cohort, it is important to highlight that some of the observed differences are in line with previous reports, in larger cohort studies, showing that Asians are at a greater risk of T2D than Caucasians due to differential fat partitioning (Lear et al., 2007; Lê et al., 2011; Nazare et al., 2012; Chen et al., 2018).

Strengths of our study include a healthy, primarily normoglycaemic, cohort from both ethnicities, in which we evaluated relationships between body fat compartments and ectopic lipid deposition in the absence of significant comorbidities. Pancreas fat determined by the MR-opsy method was averaged from the head, body, and tail of the pancreas to account for potential heterogeneity in fat distribution and was the most significant independent predictor of FPG in this cohort of women. In the absence of gold standard clamp technique to assess IR, the preferred HOMA2-IR method was used. Limitations include T1-weighting of the pancreas fat acquisition, which will overestimate the pancreatic fat, precluding direct comparison with other studies, however, they are internally consistent in comparing ethnicities. Causality cannot be confirmed from associations observed in this cross-sectional study and hence the authors are conducting longitudinal assessments in this cohort of women. Also, that these observations are from a single gender and may not be generalised to both genders due to likely gender-specific differences in energy substrate-utilisation patterns with dimorphism in glucose and fatty acid metabolism. Nonetheless, methodologies used for data collection and analyses are robust and validated.

## Data Availability Statement

De-identified data will be shared and made available upon reasonable request to the corresponding author and subject to an approved proposal and data access agreement.

## Ethics Statement

The study was reviewed and approved by the New Zealand Health and Disabilities Ethics Committee (16/STH/23). The study is registered with the Australian New Zealand Clinical Trials Registry ACTRN12616000362493. All participants provided written informed consent to participate in this study.

## Author Contributions

IS: study design and supervision, participant recruitment, data analyses and interpretation, manuscript preparation, and corresponding author. WY and LL: study design, participant recruitment, and data collection. YJ: senior statistician and advisor, data curation, and formal analysis. RM: study design, study advisor, and critical revision of manuscript. LP: study design, supervision of body composition (DXA), and critical revision of manuscript. GC: study design and methodology and critical revision of manuscript. CP: advisor MR methodology, software, and supervision. JL: development of MRI/S scan protocols and MRS processing methods. KH: supervised and reviewed MRI post-scan analysis and critical revision of manuscript. SP: funding, PI on project, study design and supervision, critical revision of manuscript, and senior author. All authors approved of the final version of the manuscript.

## Conflict of Interest

The authors declare that the research was conducted in the absence of any commercial or financial relationships that could be construed as a potential conflict of interest.

## Publisher’s Note

All claims expressed in this article are solely those of the authors and do not necessarily represent those of their affiliated organizations, or those of the publisher, the editors and the reviewers. Any product that may be evaluated in this article, or claim that may be made by its manufacturer, is not guaranteed or endorsed by the publisher.
