# Supplementary material for: Pancreas Fat, an Early Marker of Metabolic Risk? A Magnetic Resonance Study of Chinese and Caucasian Women: TOFI_Asia Study
Source: Front Physiol. 2022 Mar 31;13:819606. doi: 10.3389/fphys.2022.819606 (PMC9008457; doi:10.3389/fphys.2022.819606)

frontiers

Supplementary Material

Pancreas Fat, an Early Marker of Metabolic Risk? A Magnetic Resonance Study of Chinese and Caucasian women: TOFI_Asia study

Ivana R Sequeira[1,2], Wilson C Yip[1,2], Louise WW Lu[1,2], Yannan Jiang[3], Rinki Murphy[2,4,5,6], Lindsay D Plank[7], Garth JS Cooper[4,8,9,10] Carl N Peters[4,11], Jun Lu[12], Kieren G Hollingsworth[13], Sally D Poppitt[1,2,4,9,14].

[1]Human Nutrition Unit, Faculty of Science, School of Biological Sciences, University of Auckland, Auckland, New Zealand

[2]High Value Nutrition National Science Challenge, Auckland, New Zealand

[3]Department of Statistics, Faculty of Science, University of Auckland, Auckland, New Zealand

[4]Department of Medicine, Faculty of Medical and Health Sciences, University of Auckland, Auckland, New Zealand

[5]Auckland District Health Board, Auckland, New Zealand

[6] Maurice Wilkins Centre for Molecular Biodiscovery, University of Auckland, Auckland, New Zealand

[7]Department of Surgery, Faculty of Medical and Health Sciences, University of Auckland, Auckland, New Zealand

[8]Centre for Advanced Discovery and Experimental Therapeutics (CADET), Division of Cardiovascular Sciences, Faculty of Biology, Medicine and Health, University of Manchester, Manchester, United Kingdom

[9] Faculty of Science, School of Biological Sciences, University of Auckland, Auckland, New Zealand

[10]Department of Pharmacology, Division of Medical Sciences, University of Oxford, Oxford, United Kingdom

[11]Waitemata District Health Board, Auckland, New Zealand

[12]Faculty of Health and Environmental Sciences, Auckland University of Technology, Auckland, New Zealand

[13]Translational and Clinical Research Institute, Faculty of Medical Sciences, Newcastle University, Newcastle upon Tyne, United Kingdom

[14]Riddet Centre of Research Excellence (CoRE) for Food and Nutrition, New Zealand.

Supplementary Table 1: Relationships between fasting plasma glucose, and insulin resistance (log HOMA2-IR) with visceral adipose tissue, pancreas and liver fat in the MR cohort unadjusted, and adjusted for age, % total body fat.

		FPG (mmol/L)				log HOMA2-IR			
		**β**	**SE**	**t**	**P value**	**β**	**SE**	**t**	**P value**
**ALL**									
**Unadjusted**	Visceral adipose tissue, MR-VAT (cm^2)	0.007	0.002	4.3	<0.0001	0.004	0.002	2.00	0.05
**Adjusted**	Visceral adipose tissue, MR-VAT (cm^2)	0.003	0.003	1.15	0.25	0.002	0.003	0.73	0.47
	+Age	0.01	0.006	2.15	0.04	-0.008	0.007	-1.24	0.22
	+ TBF%	0.01	0.01	1.21	0.23	0.03	0.01	2.58	0.01
	+ Chinese	0.22	0.13	1.65	0.11	0.26	0.15	1.74	0.09
	Intercept	3.66	0.48	7.57	<0.0001	-0.90	0.54	-1.66	0.10
**Unadjusted**	Pancreas fat (%)	0.14	0.03	4.05	<0.0001	0.05	0.04	1.20	0.24
**Adjusted**	Pancreas fat (%)	0.08	0.04	1.99	0.05	0.008	0.05	0.16	0.87
	+Age	0.01	0.005	2.21	0.03	-0.007	0.006	-1.09	0.28
	+ TBF%	0.01	0.009	1.41	0.16	0.04	0.01	3.44	0.001
	+ Chinese	0.22	0.13	1.68	0.10	0.28	0.15	1.82	0.07
	Intercept	3.63	0.40	9.16	<0.0001	-1.04	0.47	-2.22	0.03
**Unadjusted**	Liver fat (%)	0.03	0.02	1.71	0.09	0.02	0.02	0.89	0.38
**Adjusted**	Liver fat (%)	-0.005	0.02	-0.30	0.77	-0.006	0.02	-0.35	0.72
	+Age	0.02	0.005	3.84	<0.0001	-0.002	0.006	-0.36	0.72
	+ TBF%	0.02	0.009	2.32	0.02	0.04	0.01	3.68	<0.0001

		FPG (mmol/L)				log HOMA2-IR			
		β	SE	t	P value	β	SE	t	P value
	+ Chinese	0.28	0.14	2.03	0.05	0.33	0.15	2.19	0.03
	Intercept	3.28	0.42	7.84	<0.0001	-1.22	0.46	-2.63	0.01
**<u>CHINESE</u>**									
**Unadjusted**	Visceral adipose tissue, MR-VAT (cm^2)	0.004	0.003	1.49	0.15	0.001	0.003	0.44	0.67
**Adjusted**	Visceral adipose tissue, MR-VAT (cm^2)	-0.001	0.004	-0.14	0.89	0.002	0.004	0.49	0.63
	+Age	0.01	0.008	1.90	0.07	-0.02	0.008	-2.28	0.03
	+ TBF%	0.02	0.03	0.91	0.37	0.02	0.03	0.80	0.43
	Intercept	3.72	0.92	4.06	<0.0001	0.24	0.96	0.25	0.81
**Unadjusted**	Pancreas fat (%)	0.10	0.04	2.36	0.03	-0.02	0.05	-0.41	0.69
**Adjusted**	Pancreas fat (%)	0.07	0.04	1.64	0.11	-0.01	0.05	-0.30	0.77
	+Age	0.01	0.007	1.72	0.10	-0.02	0.007	-2.19	0.04
	+ TBF%	0.01	0.02	0.73	0.47	0.03	0.02	1.66	0.11
	Intercept	3.89	0.70	5.53	<0.0001	-0.07	0.77	-0.09	0.93
**Unadjusted**	Liver fat (%)	0.003	0.02	0.16	0.87	-0.02	0.02	-0.81	0.42
**Adjusted**	Liver fat (%)	-0.02	0.02	-1.05	0.30	-0.03	0.02	-1.14	0.26
	+Age	0.02	0.007	2.36	0.03	-0.01	0.007	-1.99	0.06
	+ TBF%	0.03	0.02	1.51	0.14	0.04	0.02	2.0	0.06
	Intercept	3.45	0.78	4.43	<0.0001	-0.42	0.82	-0.51	0.61
**<u>CAUCASIAN</u>**									
**Unadjusted**	Visceral adipose tissue, MR-VAT (cm^2)	0.009	0.002	4.16	<0.0001	0.006	0.003	2.16	0.04
**Adjusted**	Visceral adipose tissue, MR-VAT (cm^2)	0.006	0.004	1.40	0.173	0.001	0.005	0.15	0.88

		FPG (mmol/L)				log HOMA2-IR			
		**β**	**SE**	**t**	**P value**	**β**	**SE**	**t**	**P value**
	+Age	0.009	0.01	0.96	0.34	0.001	0.01	0.13	0.89
	+ TBF%	0.009	0.01	0.67	0.51	0.04	0.02	2.35	0.03
	Intercept	3.80	0.64	5.91	<0.0001	-1.46	0.74	-1.97	0.06
**Unadjusted**	Pancreas fat (%)	0.19	0.06	3.35	0.002	0.14	0.07	1.90	0.07
**Adjusted**	Pancreas fat (%)	0.10	0.09	1.15	0.26	0.03	0.11	0.30	0.77
	+Age	0.01	0.009	1.06	0.30	0.00	0.01	-0.02	0.98
	+ TBF%	0.01	0.01	0.85	0.40	0.04	0.02	2.28	0.03
	Intercept	3.67	0.56	6.62	<0.0001	-1.37	0.66	-2.08	0.05
**Unadjusted**	Liver fat (%)	0.05	0.02	1.95	<0.0001	0.04	0.03	1.58	0.13
**Adjusted**	Liver fat (%)	0.01	0.02	0.42	0.68	0.01	0.03	0.40	0.69
	+Age	0.02	0.008	2.86	0.008	0.008	0.008	0.92	0.37
	+ TBF%	0.02	0.01	1.60	0.12	0.03	0.01	2.75	0.01
	Intercept	3.23	0.53	6.13	<0.0001	-1.57	0.56	-2.79	0.009

Data are presented as beta coefficients for each metabolic risk factor with FPG and log HOMA2-IR. Models for all women additionally also adjusted for ethnicity. Statistical significance at $P < 0.05$. Pancreas fat determined from MRI scans in 65 women (31 Caucasian and 34 Chinese); liver fat determined from MRS scans in 67 women (33 Caucasian and 34 Chinese).

Supplementary Table 2A: Stepwise linear regression models with significant independent factors that predict (i) fasting plasma glucose and (ii) insulin resistance (log HOMA2-IR) in women (n = 68) from the MR study.

	β-coefficient	SE	t	P value
FPG (mmol/L)				
Intercept	4·50	0·16	28.60	<0·0001
Pancreas fat (%)	0.14	0.03	4.05	0.0001
log HOMA2-IR				
Intercept	-1.45	0.31	-4.75	<0.0001
Chinese	0.30	0.11	2.71	0.009
DXA-%TBF	0.02	0.008	2.49	0.02
Amylin (pg/mL)	0.01	0.006	2.14	0.04
C-peptide (pg/mL)	0.001	0.0001	3.57	0.001

Summary of the Stepwise models are as follows (i) FPG: $R^2 = 0.21$, $P < 0.0001$ (ii) log HOMA2-IR: $R^2 = 0.56$, $P < 0.0001$. Models for each outcome include all independent significant predictors ($P < 0.10$); details available in Table 4. Statistical significance was set at $P < 0.05$.

Supplementary Table 2B: Least angle regression (LAR) models with significant independent factors that predict (i) fasting plasma glucose (FPG) and (ii) insulin resistance (log HOMA2-IR) in women (n = 68) from the MR study

	β-coefficient	SE	t	P value
FPG (mmol/L)				
Intercept	4·50	0·16	28.60	<0·0001
Pancreas fat (%)	0.14	0.03	4.05	0.0001
log HOMA2-IR				
Intercept	-1.25	0.31	-4.02	0.0002
DXA-%TBF	0.02	0.008	2.17	0.03
Amylin (pg/mL)	0.01	0.006	2.45	0.02
C-peptide (pg/mL)	0.0004	0.0002	3.09	0.003

Summary of the models are as follows (i) FPG: $R^2 = 0.21$, $P < 0.0001$ (ii) log HOMA2-IR: $R^2 = 0.51$, $P < 0.0001$. Models for each outcome include all independent significant predictors ($P < 0.10$); details available in Table 4. Statistical significance was set at $P < 0.05$

Supplementary Figure 1: Relationship between BMI with % pancreas fat and % liver fat using linear regression models in the entire cohort of women (open circles: 34 Caucasian, 34 Chinese) and between each ethnic group (open triangles: Caucasian; solid diamonds: Chinese). Linear regression lines and equations shown for each ethnicity for % pancreas and % liver fat

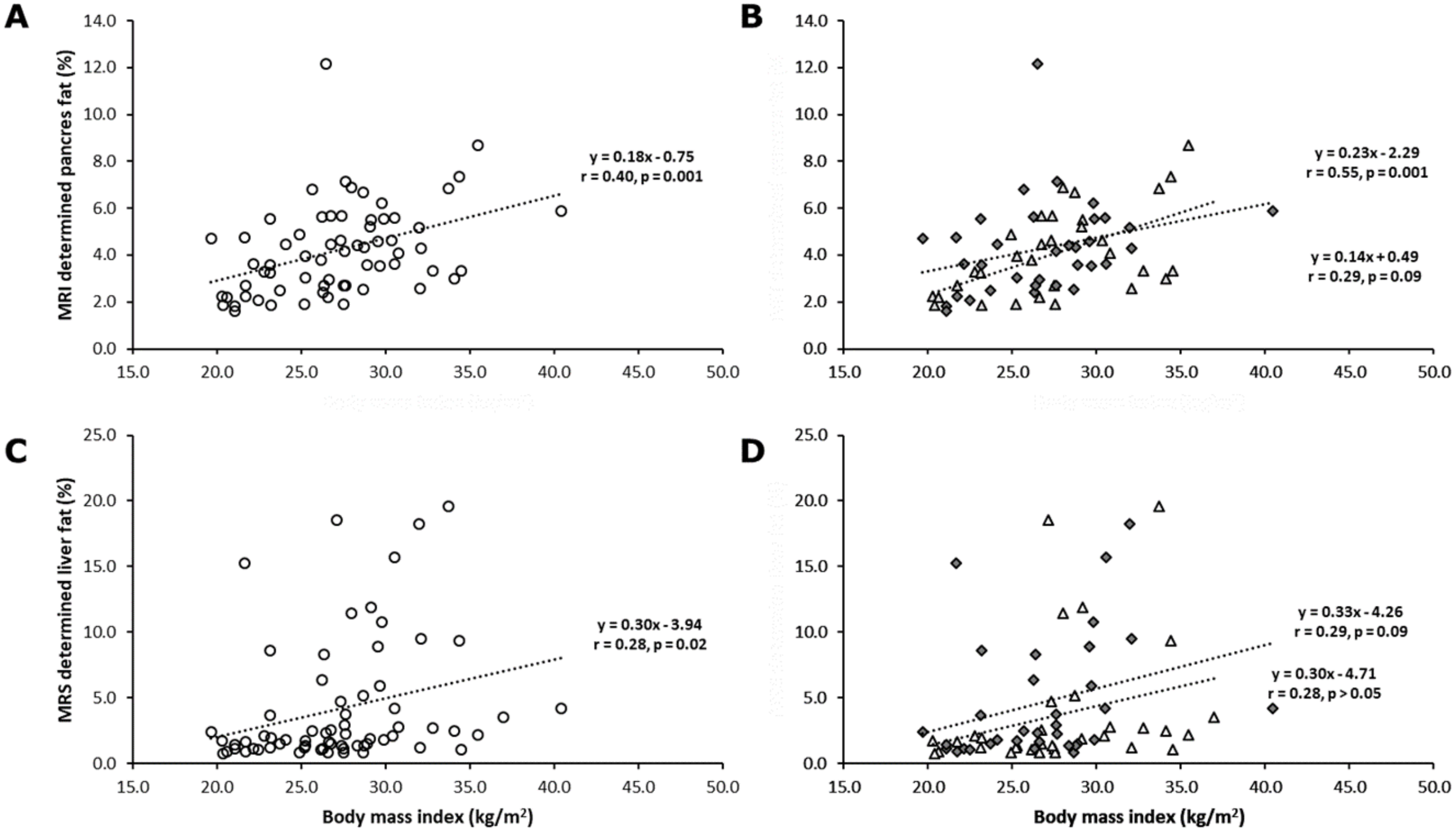

Supplementary Figure 2: Relationship between abdominal ectopic fat depots, assessed using MRI/S, with fasting plasma glucose using linear regression models in the entire cohort of women (open circles: 34 Caucasian, 34 Chinese)

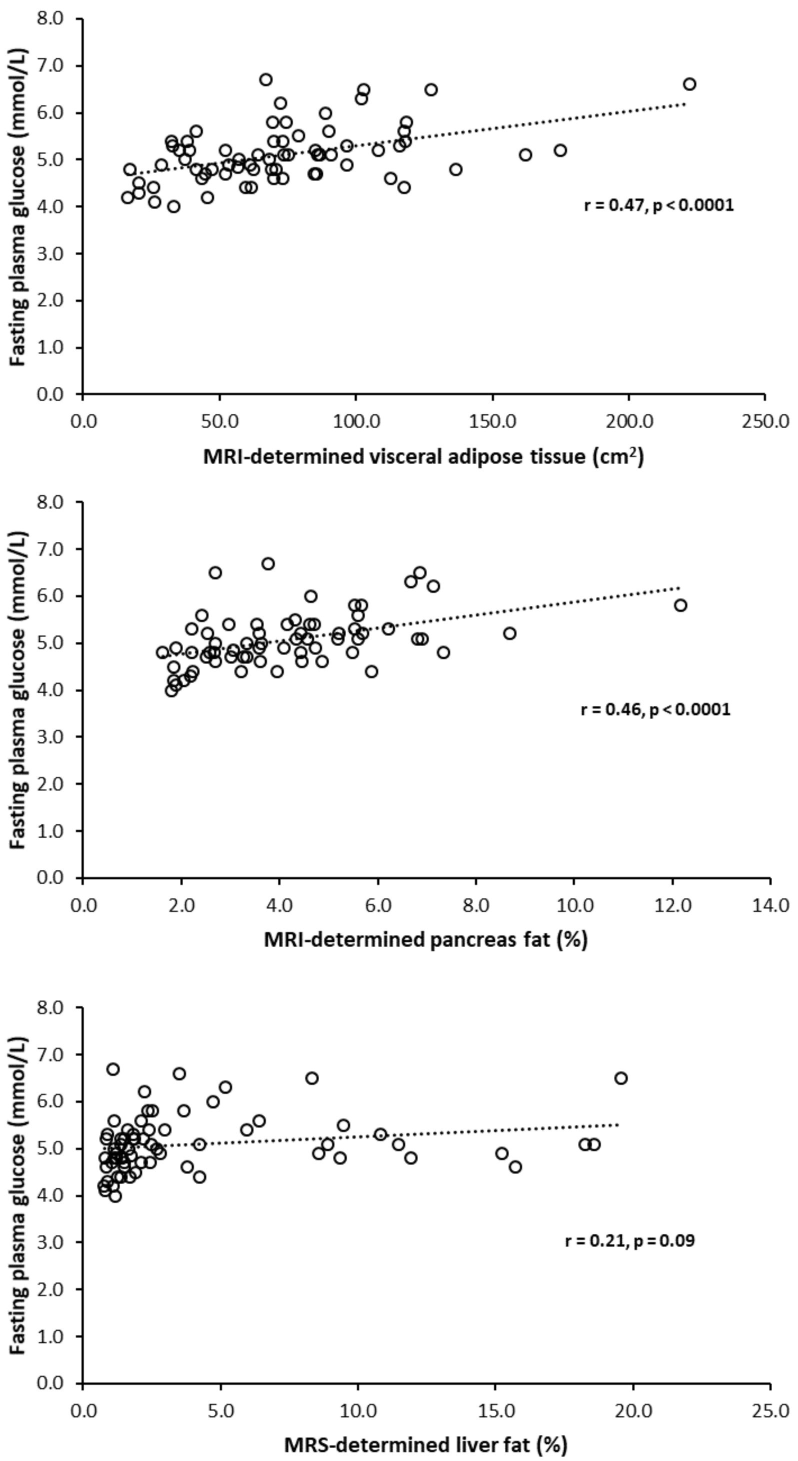

Supplement: Supplementary file 1 [file Data_Sheet_1.pdf]
